# Risk factor analysis and nomogram for predicting poor symptom control in smoking asthmatics

**DOI:** 10.1186/s12890-024-03076-9

**Published:** 2024-06-01

**Authors:** Jinxin Ma, Ziheng Chen, Ke Wu, Jiahui Lei, Limin Zhao

**Affiliations:** 1 Department of Respiratory and Critical Care Medicine, People’s Hospital of Zhengzhou University, Zhengzhou, Henan Province 450003 People’s Republic of China; 2 Department of Respiratory and Critical Care Medicine, Luoyang Central Hospital Affiliated to Zhengzhou University, Luoyang, Henan Province 471009 People’s Republic of China; 3 Department of Respiratory and Critical Care Medicine, Affiliated Hospital of Guizhou Medical University, Guiyang, Guizhou Province 550004 People’s Republic of China; 4 Department of Respiratory and Critical Care Medicine, Henan Provincial People’s Hospital, People’s Hospital of Zhengzhou University, People’s Hospital of Henan University, Zhengzhou, Henan Province 450003 People’s Republic of China

**Keywords:** Asthma, Smoking, Control level, Nomogram, Prognosis

## Abstract

**Background:**

Smoking induces and modifies the airway immune response, accelerating the decline of asthmatics’ lung function and severely affecting asthma symptoms’ control level. To assess the prognosis of asthmatics who smoke and to provide reasonable recommendations for treatment, we constructed a nomogram prediction model.

**Methods:**

General and clinical data were collected from April to September 2021 from smoking asthmatics aged ≥14 years attending the People’s Hospital of Zhengzhou University. Patients were followed up regularly by telephone or outpatient visits, and their medication and follow-up visits were recorded during the 6-months follow-up visit, as well as their asthma control levels after 6 months (asthma control questionnaire-5, ACQ-5). The study employed R4.2.2 software to conduct univariate and multivariate logistic regression analyses to identify independent risk factors for ‘poorly controlled asthma’ (ACQ>0.75) as the outcome variable. Subsequently, a nomogram prediction model was constructed. Internal validation was used to test the reproducibility of the model. The model efficacy was evaluated using the consistency index (C-index), receiver operating characteristic (ROC) curve, calibration curve, and decision curve.

**Results:**

Invitations were sent to 231 asthmatics who smoked. A total of 202 participants responded, resulting in a final total of 190 participants included in the model development. The nomogram established five independent risk factors (*P*<0.05): FEV1%pred, smoking index (100), comorbidities situations, medication regimen, and good or poor medication adherence. The area under curve (AUC) of the modeling set was 0.824(95%CI 0.765-0.884), suggesting that the nomogram has a high ability to distinguish poor asthma control in smoking asthmatics after 6 months. The calibration curve showed a C-index of 0.824 for the modeling set and a C-index of 0.792 for the self-validation set formed by 1000 bootstrap sampling, which means that the prediction probability of the model was consistent with reality. Decision curve analysis (DCA) of the nomogram revealed that the net benefit was higher when the risk threshold probability for poor asthma control was 4.5 − 93.9%.

**Conclusions:**

FEV1%pred, smoking index (100), comorbidities situations, medication regimen, and medication adherence were identified as independent risk factors for poor asthma control after 6 months in smoking asthmatics. The nomogram established based on these findings can effectively predict relevant risk and provide clinicians with a reference to identify the poorly controlled population with smoking asthma as early as possible, and to select a better therapeutic regimen. Meanwhile, it can effectively improve the medication adherence and the degree of attention to complications in smoking asthma patients.

**Supplementary Information:**

The online version contains supplementary material available at 10.1186/s12890-024-03076-9.

## Introduction

Asthma, a common chronic airway disease worldwide, is a heterogeneous clinical syndrome that interacts with a considerable number of factors, such as environment and genetics, and is prevalent worldwide [1]. The disability-adjusted life years (DALYs) of asthma in 2019 ranked eighth among 369 diseases and injuries [2, 3]. According to the China Pulmonary Health (CPH) study, the prevalence of asthma among people over the age of 20 in China was approximately 4.2%, which is higher than the global prevalence [4]. However, the diagnosis rate of asthma in China is only 28.8%, and only 39.2% of patients achieve clinical control [4, 5].

Asthma risk factors included obesity, cigarette, genetic factors, air pollution, gender, occupational exposure, microenvironment, and vitamin D deficiency. Smoking (9.9%) was second only to obesity (16.9%) among the various factors affecting asthma DALYs in 2019 [3]. Jaakkola [6] et al. found that smoking was associated with decreased lung function with new-onset asthma and there was a dose-response relationship, meanwhile can accelerate the decline of lung function in patients with asthma [7].

Since the proposal of the concept of ‘asthma control’ by the Global Initiative for Asthma (GINA) in 2006, ‘symptom control’ has been used as a measure to assess asthma and determine treatment options. It found that the mean asthma control test (ACT) score was about 1 point lower in combustible tobacco users compared with people who had never used tobacco (22.4 (SE=0.1) vs 23.6 (SE=0.2), *P*<0.01) [8].China is the world’s largest tobacco victim country - the smoking rate of people aged ≥15 years is as high as 26.6%, and the smoking rate of asthma patients is similar to that of ordinary people, which is 20-25%. However, in order to control for the influence of confounding factors and other potential mechanisms, a quantity of previous studies have excluded smoking asthma patients, resulting in a lack of progress in related studies on smoking asthma patients and a lack of targeted treatment guidance programs. The aim of this study was to explore the risk factors for smoking-related asthma, to determine the relationship between these risk factors and poor symptom control, and to develop and validate a model for predicting the risk of poor asthma control in such patients after 6 months.

## Materials and methods

### Participants and data extraction

This study recruited smoking asthmatics over the age of 14 who underwent lung function testing at People’s Hospital of Zhengzhou University between April and September 2021. The diagnosis of asthma was in line with GINA [9] Individuals who have smoked at least one cigarette per day for a duration exceeding six months were considered smokers. The exclusion criteria comprised the following: (1) co-existing major clinical lung diseases, other systemic serious diseases, or malignant tumors; (2) had a respiratory tract infection within the past four weeks; (3) used special types of tobacco products, such as electronic cigarettes or hookahs; (4) pregnancy or lactating; (5) unable to comprehend or disagree with the contents and problems of investigation and follow-up; and (6) changes in smoking status during follow-up.

**Fig. 1:**
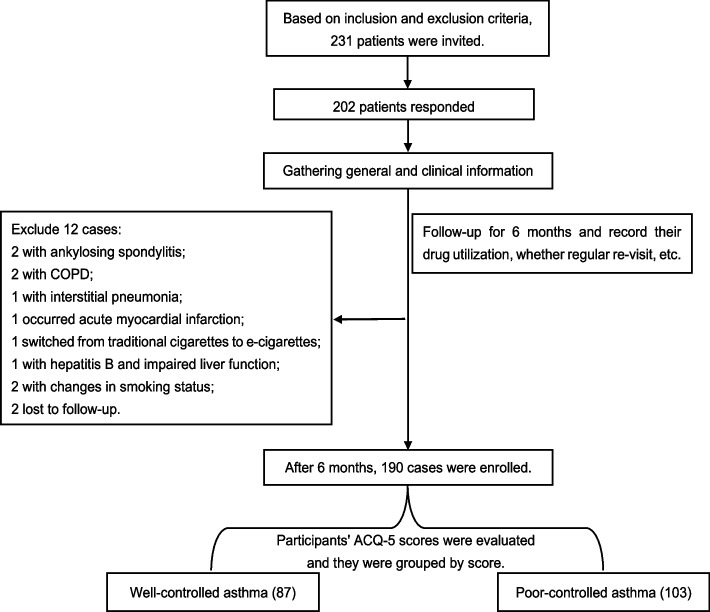
Flow chart of patient selection and group

The following general and clinical information about the patient was collected through a combination of medical records and outpatient consultations with parallel dual entry. The patients’ age, gender, weight, height, body mass index (BMI), and body surface area (BSA), occupation, educational status, smoking history, family history of asthma, duration of asthma course, good or poor medication adherence, occurrence of acute asthma exacerbations in the past 6 months, presence of allergens, and comorbidities such as allergic rhinitis, rhinitis/sinusitis, anaphylactic conjunctivitis/allergic dermatosis, obstructive sleep apnea syndrome (OSAS), hypertension, coronary atherosclerotic heart disease (CHD), gastroesophageal reflux disease (GERD), anxiety/depression; pulmonary function parameters included: forced vital capacity (FVC), forced expiratory volume in one second (FEV1) and FEV1 percentage to predicted value (FEV1%pred), and FEV1/FVC%. During the 6-month follow-up period, the patients’ drug utilization, whether regular review and ACQ-5 scores after 6 months were recorded. The data collection process is illustrated in Fig. 1.

### Definitions of predictor variables

To explore the relationships between various patient characteristics and the outcome variable, we categorized patients into subgroups based on educational status, occurrence of acute asthma exacerbations in the past 6 months, quitting smoking or not, comorbidities situations, and medication regimen, and other factors. Detailed definitions of variables are summarized below.

#### Smoking index (100)

In contrast to previous literature using pack-year, to ensure the relationship between symptom control and smoking intensity is accurately reflected, we use the ‘smoking index (100)’ to represent the amount of smoking in patients. This is calculated by multiplying number of cigarettes per day by the years of smoking and dividing by 100 [10–12].

#### Acute attack

This refers to the abrupt onset of characteristic asthma symptoms, such as chest tightness, shortness of breath, and dyspnea, necessitating emergency or hospitalization treatment, or oral or intravenous glucocorticoids and other medications.

#### Comorbidities situations

Allergic rhinitis, allergic conjunctivitis/skin disease, rhinitis/sinusitis and ten other comorbidities closely related to symptom control of asthma were collected and classified as present or absent. If comorbidities are present and are treated in accordance with medical advice or guidelines, then “comorbidities present and treated”, otherwise “comorbidities present and untreated”. “None” indicates that the patient has none of the above comorbidities.

#### Medication adherence

**Fig. 2:**
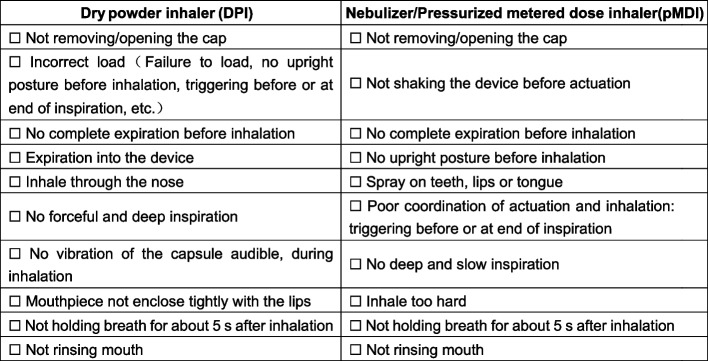
Common misuse of inhalation drug delivery devices

Two physicians with at least 5 years of clinical experience evaluated the patients’ medication adherence according to the following criteria. Patients were deemed to have “good medication adherence” if they used at least 80% of the prescribed amount of asthma medication per month and used inhalation medication devices in a standardized manner [13], otherwise they were considered to have “poor medication adherence”. Integration of clinical practice and previous research [14, 15], the presence of any of the following (Fig. 2) incorrect operations was defined as inappropriate use of an inhaled drug device.

#### Medication regimen

The term “None” means that the patient was diagnosed with asthma and did not use medication despite the appropriate treatment plan given by the physician.

### Subgroups and outcome variables

Two physicians assessed the patients’ ACQ-5 scores at month 6 after the initial visit and categorized them into “well-controlled asthma control” (ACQ-5≤0.75) and “poor-controlled asthma” (ACQ-5>0.75) groups based on their mean scores [9, 16]. The poor symptom control of asthmatics who smoke after 6 months was used as the outcome variable.

### Statistical analysis

R4.2.2 software was applied to statistically analyze the data. Univariate analysis was performed on each variable to obtain risk factors related to “poor asthma control” (*P*<0.05) as the outcome variable. The significant variables obtained from the above analysis were subjected to multivariate logistic regression using the backward stepwise regression method to obtain predictors that could be included in the prediction model. The performance and resolution of the model were assessed using the receiver operating characteristic (ROC) curve and the area under the curve (AUC). The final nomogram is plotted and internally validated using a 1000 bootstrap resampling technique to test the stability of the prediction model. The C-index was calculated using the Concordance statistic (C statistic) to assess the model’s accuracy. The clinical utility of the nomogram was assessed by plotting calibration and decision curves. Statistical analysis was performed by two-sided tests, and a significant difference was defined as *P*<0.05.

## Results

### Participant characteristics

**Fig. 3:**
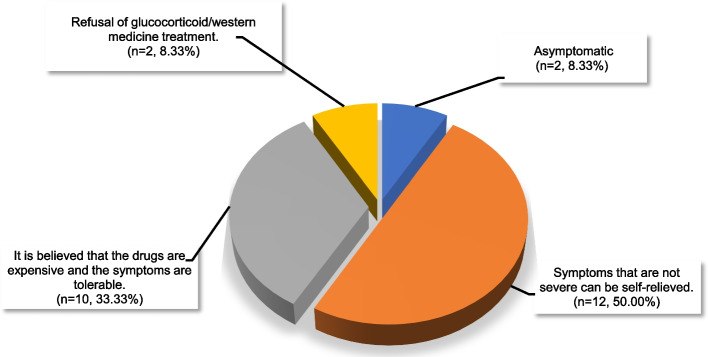
Specific reasons and percentage of the 24 patients who did not take any medication

For this study, invitations were sent to 231 patients, and 202 responded, resulting in a response rate of 87.45%. A total of 190 cases with valid case data were included in the study. Of these, 87 cases exhibited well-controlled asthma, while 103 cases showed poor control of the condition. Out of the 190 patients diagnosed with asthma, 106 (55.79%) had poor medication adherence. Among them, 24 (12.70%) were not using asthma medication, and Fig. 3 shows the specific reasons why this occurs. Additionally, regular review could only be conducted for 61 cases (32.11%). Table 1 summarized the demographic and clinical characteristics of these participants: age, educational status, with occupational exposures or not, any acute attack within 6 months, smoking index (100), FEV1, FEV1pred, FEV1%pred, FEV1/FVC%, combined hypertension, comorbidities situations, whether regular review, medication regimen, and good or poor medication adherence were significantly different between the two groups (*P*<0.05). And these factors were analyzed by univariate analysis (Supplementary table 1).

**Table 1:** Demographic and clinical characteristics of asthmatics who smoke

**Characteristics**	**Total (*n*=190)**	**Well-controlled asthma** **(*n*=87)**	**Poor-controlled asthma** **(*n*=103)**	***P* value**
**Age (years)**	44.50(31.00, 55.00)	41.00(29.00, 51.50)	49.00(34.00, 57.00)	0.004
**Gender**				0.757
Male	179(94.21%)	81(93.10%)	98(95.15%)	
Female	11(5.79%)	6(6.90%)	5(4.85%)	
**High (cm)**	170.00(167.13, 175.00)	172.00(167.50, 175.50)	170.00(167.25, 173.75)	0.195
**Weight (kg)**	74.27(11.54)	73.34(12.34)	75.05(10.82)	0.316
**BMI (kg/m**^**2**^**,)**	25.43(3.62)	24.92(3.59)	25.86(3.60)	0.074
**BSA (m**^**2**^**)**	1.86(0.14)	1.85(0.16)	1.86(0.13)	0.624
**Occupational exposures**				<0.001
No	84(44.21%)	50(57.47%)	34(33.01%)	
Yes	106(55.79%)	37(42.53%)	69(66.99%)	
**Educational status**				<0.001
Middle school or less	90(47.37%)	29(33.33%)	61(59.22%)	
High school	40(21.05%)	18(20.69%)	22(21.36%)	
College and higher	60(31.58%)	40(45.98%)	20(19.42%)	
**Initial diagnosis**				0.190
No	82(43.16%)	33(37.93%)	49(47.57%)	
Yes	108(56.84%)	54(62.07%)	54(52.42%)	
**Any acute attack within 6 months**				0.027
No	145(76.32%)	73(83.91%)	72(69.90%)	
Yes	45(23.68%)	14(16.09%)	31(30.10%)	
**Smoking index (100)**	1.70 (0.43, 4.00)	1.10 (0.32, 2.80)	2.25 (1.00, 5.00)	<0.001
Quitting smoking or not*				0.679
No	125(65.79%)	59(67.81%)	66(64.07%)	
Quitting	20(10.53%)	10(11.49%)	10(9.71%)	
Successful quitter	45(23.68%)	18(20.69%)	27(26.21%)	
**Family history**				0.757
No	179(94.21%)	81(93.10%)	98(95.15%)	
Yes	11(5.79%)	6(6.90%)	5(4.85%)	
**Allergens**				0.880
No	70(36.84%)	33(37.93%)	37(35.92%)	
Yes	120(63.16%)	54(62.07%)	66(64.08%)	
**Allergic rhinitis**				0.660
No	107(56.32%)	47(54.02%)	60(58.25%)	
Yes	83(43.68%)	40(45.98%)	43(41.75%)	
**Rhinitis/sinusitis**				0.643
No	169(88.95%)	76(87.36%)	93(90.29%)	
Yes	21(11.05%)	11(12.64%)	10(9.71%)	
**Anaphylactic conjunctivitis /allergic dermatosis**				0.449
No	173(91.05%)	81(93.10%)	92(89.32%)	
Yes	17(8.95%)	6(6.90%)	11(10.68%)	
**OSAS**				0.517
No	180(94.74%)	81(93.10%)	99(96.12%)	
Yes	10(5.26%)	6(6.90%)	4(3.88%)	
**Hypertension**				0.049
No	159(83.68%)	78(89.66%)	81(78.64%)	
Yes	31(16.32%)	9(10.34%)	22(21.36%)	
**CHD**				0.349
No	180(94.74%)	84(96.55%)	96(93.20%)	
Yes	10(5.26%)	3(3.45%)	7(6.80%)	
**GERD**				0.190
No	180(94.74%)	80(91.95%)	100(97.09%)	
Yes	10(5.26%)	7(8.05%)	3(2.91%)	
**Anxiety/depression**				0.388
No	177(93.16%)	83(95.40%)	94(91.26%)	
Yes	13(6.84%)	4(4.60%)	9(8.74%)	
**FEV1**	3.15 (2.67, 3.53)	3.32 (2.91, 3.82)	3.00 (2.50, 3.39)	<0.001
**FEV1pred**	3.46 (3.06, 3.91)	3.54 (3.18, 3.99)	3.38 (2.99, 3.74)	0.023
**FEV1%pred**	91.17 (84.34, 96.96)	94.18(88.25, 100.80)	88.18(80.99, 93.10)	<0.001
**FEV1/FVC%**	71.05 (66.53, 76.95)	74.30 (70.01, 78.87)	68.72 (64.89, 73.07)	<0.001
**Comorbidities situations**				0.005
Present and treated	52(27.37%)	15(17.24%)	37(35.92%)	
Present and untreated	78(41.05%)	45(51.72%)	33(32.04%)	
None	60(31.58%)	27(31.03%)	33(32.04%)	
**Regular review**				<0.001
No	129(67.89%)	48(55.17%)	81(78.64%)	
Yes	61(32.11%)	39(44.83%)	22(21.36%)	
**Medication regimen**				<0.001
None	24(12.63%)	2(2.30%)	22(21.36%)	
ICS+LABA	95(50.0%)	46(52.87%)	49(47.57%)	
ICS+LABA+LTRA	69(36.32%)	38(43.68%)	31(30.10%)	
LTRA	2(1.05%)	1(1.15%)	1(0.97%)	
**Medication adherence**				<0.001
Poor	106(55.79%)	32(36.78%)	74(71.84%)	
Good	84(44.21%)	55(63.22%)	29(28.16%)	

Continuous variables that meet the normal distribution are expressed as mean (standard deviation), otherwise as median (interquartile range); categorical variables are expressed as frequency (percentage)

^*^As per the WHO and previous research, a “quitter” refers to someone who smoked daily for at least six months but has now ceased smoking at the time of the survey. A person who has stopped smoking for over 2 years at the time of the survey is deemed a “successful quitter,” whereas those who have quit smoking for less than 2 years are referred to as “quitting” [63, 64]

*OSAS* obstructive sleep apnea syndrome, *CHD* coronary atherosclerotic heart disease, *GERD* gastroesophageal reflux disease, *ICS* inhaled corticosteroids, *LABA* long-acting β2 agonists, *LTRA* leukotriene receptor antagonist

### Screening the best predictors and validating their plausibility

**Fig. 4:**
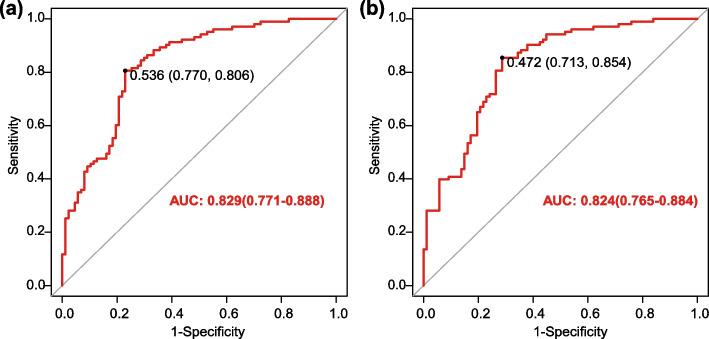
ROC curve of nomogram for predicting the risk of poor asthma control in smoking asthmatics after a 6-month period. **a** 6 predictors including FEV1pred; **b** 5 predictors after excluding FEV1pred

The 14 risk factors screened by univariate analysis (*P*<0.05) were included in a backward multiple regression analysis, and 6 relevant variables were obtained (Table 2). AUC was determined to be 0.829 (95% CI 0.771-0.888), incorporating all 6 variables in the analysis, as depicted in Fig. 4a of this study. 5 variables, excluding FEV1pred (*P*>0.05), were utilized to construct the ROC curve (Fig. 4b), yielding an AUC of 0.824 (95% CI 0.765-0.884). A differential analysis was performed on two ROC curves, and the p-value was greater than 0.05, demonstrating that the two models had comparable efficacy. In order to optimize model simplicity and ensure consistency, five relatively independent risk factors were ultimately screened: FEV1%pred, smoking index (100), comorbidities situations, medication regimen, and good or poor medication adherence.

**Table 2:**
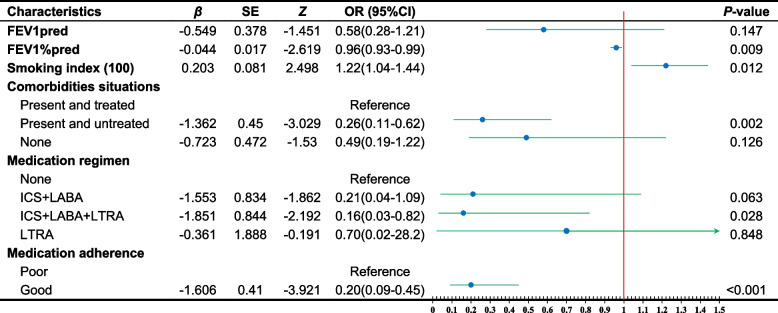
Multivariate logistic regression analysis to screen the best predictors

### Nomogram development and validation

**Fig. 5:**
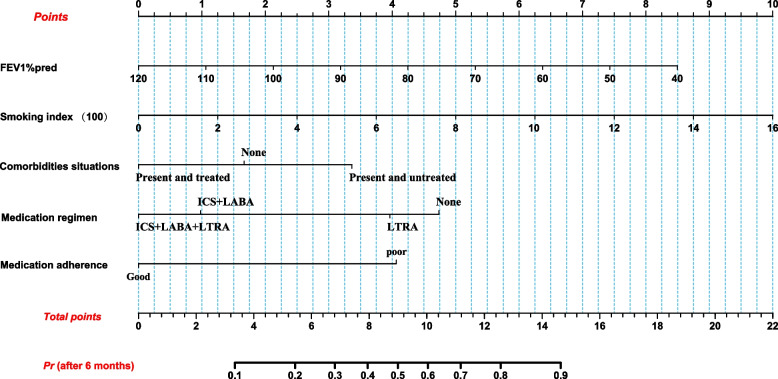
Nomogram for predicting the risk of poor asthma control in smoking asthmatics after a 6-month period. The sum of the individual scores for each factor is the total score, and the corresponding of poor asthma control risk is obtained by making a vertical line across the total score points

The 5 predictors obtained from the aforementioned screening were integrated into R4.2.2 to generate the nomogram prediction model, as shown in Fig 5. Each risk factor is associated with a scale mark that represents the range of values that can be taken for that factor. The length of the line reflects the magnitude of the factor’s contribution to the outcome variable. The individual scoring scale at the top of the image indicates the corresponding scores of different values of the risk factors, and the sum of the individual scores of each factor is the total score. A vertical line is drawn across the total score points to obtain the corresponding “risk probability” at the bottom. For instance, a patient with asthma who has been smoking 40 cigarettes per day for 30 years (smoking index (100) score≈7.5), has hypertension and allergic rhinitis without regular treatment (comorbidities situations score≈3.375), and has an FEV1%pred of 70% (score≈5.35). If this patient is regularly treated (medication adherence≈0)with ICS+LABA+LTRA regimen (medication regimen≈0) for the next 6 months, his total score is 16.225 and the risk of poor asthma symptom control after 6 months is greater than 90%. The patient’s poor symptom control is attributed to excessive smoking and irregular treatment of comorbidities. Therefore, the patient should be educated on the importance of smoking cessation and regular use of medication to treat comorbidities. To mitigate the high risk of poor asthma control, the dose of asthma medication can be appropriately increased or adjunctive medication can be added.

**Fig. 6:**
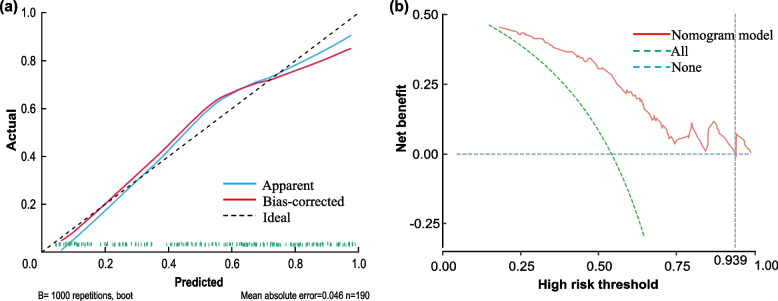
The validation of Nomogram. **a** Calibration curves constructed by bootstrap validation. The apparent and bias-corrected curves exhibit satisfactory conformity with the reference line, albeit exhibiting minor divergences from it. **b** The DCA curve of the nomogram which predict the risk of poor asthma control in smoking asthmatics

Calibration curve of the training set is displayed in Fig. 6(a), with the bootstrap method used to construct the curve after bias corrections. The apparent(C-index=0.824) and bias-corrected(C-index=0.792) curves exhibit satisfactory conformity with the reference line, which mean the model demonstrates high precision in prediction. As can be seen from the decision curve in Fig. 6(b), the model exhibits a satisfactory positive net benefit when the threshold probability of poor asthma control in smoking asthmatics after 6 months ranges from 4.5 to 93.9. clinical benefit and utility. These results suggest that the model the model holds significant promise for clinical benefit and utility.

## Discussion

Smoking is the second most significant risk factor (9.9%) for reducing the lifespan of asthma patients, amongst many other factors [3]. Compared with non-smokers, patients with smoking asthma experience poorer symptom control, a higher frequency of acute attacks, a more rapid decline in lung function, less effective ICS treatment, and a lack of guidance on a more efficient medication regimen, imposing a considerable burden on both patients and society [8, 9, 17]. In this study, we collected data from 190 patients suffering from smoking-asthma to explore the factors that could impact the level of symptom control after a 6 month period. Additionally, we developed and validated a nomogram to forecast their likelihood of experiencing inadequate symptom control after six months. To our knowledge, this instrument is pioneering in prognosticating the prognosis of smoking-asthma, which could enhance clinicians’ capability to detect Potential population with inadequate symptom control.

We have identified five predictors to construct the nomogram, which comprise FEV1%pred, smoking index (100), comorbidities situations, medication regimen, and whether medication adherence good or poor. Asthma sufferers exhibit varying degrees of lung function damage. Fielding [18] et al. found that a 10% reduction in%FEV1 between baseline and 3months was associated with 21% increased odds for having poor asthma control (95% CI 0-45) 6 months after baseline; even within the “normal range” (80-120%), FEV1%pred is still associated with increased risk of late asthma exacerbation and uncontrolled asthma. Smoking in asthmatic patients can exacerbate the decline of lung function indicators, including FEV1 % pred and FEV1, and this deterioration is correlated with the amount of smoking [6, 19, 20], whereas FEV1%perd can be improved with asthma control [21]. Therefore, dynamic detection of changes in FEV1%pred can effectively reflect the impairment of lung function and the level of asthma control in asthmatics who smoke. In the present study, FEV1%pred(OR=0.96, 95%CI 0.93-0.99) was identified as an independent risk factor for poor symptom control in asthmatic smokers with an inverse relationship, further corroborating its role in risk assessment for poor asthma control in smoking. Compared with bronchial provocation test and bronchodilator test, the measurement of FEV1%pred does not require specialized personnel and drug application, which makes it more practical in monitoring changes in condition at home [22]. Similarly, it can facilitate the assessment, monitoring and treatment of asthma conditions in underdeveloped areas.

Chemicals released by cigarette smoke(CS) the risk of various ailments, including but not limited to asthma, chronic obstructive pulmonary disease (COPD), lung cancer, and coronary heart disease [23]. The tracheal epithelium serves as the primary defense line against foreign invasion. However, exposure to CS can compromise this function through decreased transepithelial resistance, increased permeability, and inhibition of tight junctions and adherens junctions formation [24, 25]. It can also reduce the allergen threshold through oxidative stress suppressing PI3K-δ/Akt pathway, activating mTOR pathway, inducing cellular senescence and other mechanisms, thereby leading to an increase in IgE and augmenting Th2-type immune responses, and ultimately increasing the risk of asthma [25–28]. In addition, CS containing high concentrations of reactive oxygen species can activate airway epithelial cells to synthesize and release inflammatory mediators such as interleukin (IL)-6 and IL-8, as well as recruit and activate neutrophils and macrophages, resulting in increased neutrophils in lung tissue [25, 29–31]. Furthermore, it can also promote the further production of oxygen free radicals in the airway epithelium of asthmatic patients, aggravate original airway injury, lead to a higher number of inflammatory cells, more severe ciliary detachment, and thicker bronchial smooth muscle compared to non-smokers, ultimately accelerating airway remodeling [25, 32]. As a result, asthmatics who smoke generally have poorer symptom control than nonsmokers, and there is a dose-response relationship. Smoking index (100) was positively correlated with poor asthma control(OR=1.22, 95% CI 1.04-1.44) in this study , and when combined with the characteristics of the high number of deaths per year due to tobacco and the high prevalence of smoking among male in China [33, 34], smoking cessation is essential to improve the prognosis of asthma patients who are smokers. Research has shown that asthmatic individuals who cease smoking for over a year experience a reduced rate of decline in their lung function compared to their previous state [6]; the promulgation of smoke-free policies in public places and private places can also reduce the emergency hospitalization rate of asthma and the prevalence of respiratory diseases in children which was found by Radó et al [35]. Therefore, strengthening smoking cessation education for asthmatic smokers, along with efficacious cessation aids, ought to be the cornerstone of asthma management in China. This study’s univariate analysis did not find any association between smoking cessation and poor asthma control. However, limitations such as the single-center data source, small sample size, varying smoking cessation time and large dispersion may have impacted the results. Further studies are needed to expand the sample size to determine the relationship between the two.

The treatment of “smoking-asthma” related comorbidities should not be neglected. An analysis with comorbidities presented and untreated as a control found that no comorbidities (OR=0.49, 95%CI 0.19 to 1.22), and comorbidities present and treated (OR=0.26, 95%CI 0.11 to 0.62) were beneficial to the control of smoking-asthma symptoms, which is in line with conventional knowledge. However, having comorbidities and treating them was more favorable for controlling smoking-asthma symptoms than those without comorbidities (OR 0.26 vs. 0.49). One possible explanation for this finding is that patients with comorbidities or underlying diseases may demonstrate increased awareness and vigilance towards their health condition. Another possible explanation may be that the comorbidities of interest in this study are intricately linked to the prognosis of asthma and display some common pathogenic mechanisms. For example, asthma, allergic rhinitis and sinusitis are all related to allergen exposure, with airway hyperresponsiveness, eosinophilia and upper airway remodeling [36–38]; smoking-asthma is non-Th2 type asthma [25], IL-17-neutrophil axis, foam cells, mast cells are involved in the development of non-Th2 type asthma, hypertension, atherosclerosis [39–43], and there is even a strong gene overlap between these diseases [44, 45]; treatment of OSAS can improve asthma symptoms, which may be related to increased neutrophil airway inflammation in OSAS patients and airway hyperresponsiveness caused by pharyngeal collapse to vagal nerve stimulation [46, 47]; GERD may induce asthma by increasing vagus nerve excitability, gastric reflux into the airways causing airway hyperresponsiveness, altering the airway microenvironment and altering epithelial gene expression [47–49], smoking may induce or exacerbate gastroesophageal reflux, and the two complement each other. In conclusion, an in-depth study of the relationship between asthma and the comorbidities with which it shares a common pathogenic mechanism may be a new idea for the development of novel drugs for asthma.

Currently, there is a lack of large-scale clinical cohort studies and guidelines on clinical medication regimens for smoking asthma. Exposure to CS can exacerbate airway inflammation in pre-existing asthma, and can also contribute to increased neutrophils and macrophages in lung tissues and decreased histone deacelytase2 (HDAC2) in the airways [28], which leads to airway inflammation in the direction of a non-Th2 phenotype and ultimately ICS resistance [25, 50]. CS can also increase the particle size of ICS, reduce its airway deposition rate, and reduce the sensitivity of the airway to ICS again [51]. However, ICS remains a preferred initial therapy for asthmatic who smoke due to its ability to mitigate inflammatory airway response, improve lung function decline, and impede airway remodeling [52, 53]. Considering the side effects of prolonged and massive application of ICS, GINA recommends that ICS combined with LABA should be prioritized over increasing ICS doses for the treatment of steroid-resistant asthma [9]. However, clinical dosing regimens for medium or severe smokers with asthma still lack large clinical cohort studies to prove their effectiveness. Asthma and COPD belong to the same category of airway obstructive diseases, and smoking asthmatics, especially those who smoke more than 10 pack-year, are phenotypically similar to COPD patients to some extent [54], so long-acting cholinergic receptor antagonists (LAMA) can be considered for the treatment of smoking-asthma. Simultaneously, there is a study that has also demonstrated that the combination of ICS, LABA, and LAMA is superior to ICS combined with LABA [55], but there is currently insufficient data from large-scale clinical studies to fully support this finding. Arachidonic acid metabolites and transcripts of enzymes such as prostaglandins and leukotrienes, which are involved in lipoxygenase and cyclooxygenase pathways, are increased in smoking asthmatics compared to nonsmokers, so LTRA may have a greater benefit in smoking asthmatics. A study by Price [56] et al. conducted a study which found that asthmatics who smoked ≥11 pack-year showed greater benefits from LTRA treatment. Monotherapy with LTRA should only be considered in certain special cases, as neither GINA nor the findings of this study suggest its use as a first-line treatment. This is supported by the better outcomes demonstrated by a combination of ICS, LABA and LTRA in managing smoking-related asthma, as shown in this study. CpG oligodeoxynucleotides and budesonide synergistically alleviated the Th17/Th2 imbalance in CS-associated asthmatic mice by modulating IL-23 secretion and blocking TSLP, but whether CpG oligodeoxynucleotides alone still have the above effects and whether the drugs can be extended to human applications requires further study [57].

The low rate of asthma control is not only related to the many risk factors, but also to poor patient compliance and inhaler technology due to the nature of attacks and remissions. A British study shows that if asthma doesn’t interfere with their normal lives, patients often don’t see the need to take daily medication for it [58]. An online survey in eight Asian countries and regions found that 90% of people overestimated their condition and thought their asthma was under control, while 50% actually had uncontrolled asthma [59]. Pressurized metered dose inhalers (pMDIs), dry powder inhalers (DPIs) and nebulizers, among other devices, each have their own merits and drawbacks. The incorrect usage of an inhaler may result in a reduction in the drug’s effective delivery rate to the airway.

In conjunction with this and previous studies, the most frequent incorrect practices included inadequate coordination of actuation and inhalation, insufficiently forceful and deep inspiration, and inadequate breath-holding post-inhalation or a breath-hold period that was too brief when utilizing pMDIs. Additionally, incomplete exhalation before inhalation, inadequate breath-holding post-inhalation, or a breath-hold period that was too brief were among the most prevalent improper behaviors when employing DPIs. The patients who had poor medication adherence in this study were as high as 55.79%, which shows that the popularization of asthma, patient education, guidance on inhalation techniques, and regular checkups still need to be strengthened.

The study outcomes demonstrated that the model exhibited a degree of sensitivity, specificity, and goodness of fit. The predictors were derived through simple queries and general screening. The nomogram transforms complex regression equations into clear and concise graphs, allowing clinicians to effectively educate their patients. This tool aids in the early identification of the at-risk group for smoking asthma with inferior symptom control. Additionally, it judges the contribution of various factors leading to poor control of the symptoms of the patients and provides focused warning signs to enhance patient compliance. Clinicians can select an appropriate treatment plan by drawing on their clinical expertise and taking into account the patient’s unique clinical circumstances. Advancing such interventions proactively contributes to the patient’s overall prognosis.

There are also limitations to this study. (1) Data collection such as electronic medical records and self-reported ACQ-5 questionnaires may be subject to information bias. However, the applicability of the ACQ questionnaire has been confirmed in several studies and its utility has been recommended by GINA [9, 60–62]. The information collected has also been verified through the medical record system and the family side, so as to minimize the information bias. (2) In this study, patients’ ACQ-5 questionnaire scores were assessed from October to April, mainly during the autumn and winter months. The distribution of allergens in the environment is different from that in spring and summer, resulting in possible differences in symptom control performance in patients with specific seasonal allergens. It is necessary to change the season of return visits and compare the model across seasons to observe any seasonal differences. (3) The data source for this nomogram was single-center and lacked a large sample size. To avoid overfitting, some factors related to asthma symptom control were not included in the model. Although the internal validation showed that the predictive model has good accuracy, it is still necessary to increase the sample size and conduct external validation to improve and adapt the model for practical application.

## Conclusion

Poor asthma symptom control after 6 months in patients with smoking asthma was associated with FEV1%pred, smoking index (100), comorbidities situations, medication regimen, and good or poor medication adherence. According to the weight of each factor in poor prognosis, an individualized treatment and control plan can be drawn up. A nomogram based on the above results can be effective for initial risk prediction.

### Supplementary Information

Supplemenetary Material 1.

## Data Availability

All data generated or analyzed during this study can be obtained from the corresponding author if justified.

## Abbreviations

ACQ-5: Asthma Control Questionnaire-5
C-index: Concordance Index
ROC: Receiver Operating Characteristic
AUC: Area Under Curve
DCA: Decision curve Analysis
DALYs: Disability-adjusted Life Years
CPH: China Pulmonary Health
GINA: Global Initiative for Asthma
ACT: Asthma Control Test
BMI: Body Mass Index
BSA: Body Surface Area
OSAS: Obstructive Sleep Apnea Syndrome
CHD: Coronary Atherosclerotic Heart Disease
GERD: Gastroesophageal Reflux Disease
FVC: Forced Vital Capacity
FEV1: Forced Expiratory Volume In One Second
FEV1%pred: FEV1 percentage to predicted value
C statistic: Concordance Statistic
ICS: Inhaled Corticosteroids
LABA: Long-acting β2 Agonists
LTRA: Leukotriene Receptor Antagonist
CS: Cigarette Smoke
COPD: Chronic Obstructive Pulmonary Disease
IL: Interleukin
HDAC2: Histone Deacelytase2
LAMA: Long-acting Cholinergic Receptor Antagonists
pMDIs: Pressurized Metered Dose Inhalers
DPIs: Dry Powder Inhalers
